# The short-term effectiveness and safety of second-generation patellofemoral arthroplasty and total knee arthroplasty on isolated patellofemoral osteoarthritis: a systematic review and meta-analysis

**DOI:** 10.1186/s13018-021-02509-z

**Published:** 2021-06-02

**Authors:** Chengxin Li, Zhizhuo Li, Lijun Shi, Fuqiang Gao, Wei Sun

**Affiliations:** 1 Department of Orthopedics, Peking University China-Japan Friendship School of Clinical Medicine, 2 Yinghuadong Road, Chaoyang District, Beijing, 100029 China; 2 Department of Orthopedics, Graduate School of Peking Union Medical College, China-Japan Friendship Institute of Clinical Medicine, 2 Yinghuadong Road, Chaoyang District, Beijing, 100029 China; 3 Beijing Key Laboratory of Immune Inflammatory Disease, China-Japan Friendship Hospital, Peking Union Medical College, 2 Yinghuadong Road, Chaoyang District, Beijing, 100029 China

**Keywords:** Second-generation patellofemoral arthroplasty, Total knee arthroplasty, Isolated patellofemoral osteoarthritis

## Abstract

**Background:**

We aimed to compare second-generation patellofemoral arthroplasty (2G PFA) with total knee arthroplasty (TKA) in treating isolated patellofemoral osteoarthritis (PFOA) by assessing the percentages of revisions, complications, and patient-reported outcome measures (PROMs).

**Methods:**

Studies that compared the outcomes of 2G PFA and TKA in the treatment of isolated PFOA were searched in electronic databases, including MEDLINE, Embase, and Web of Science. Two researchers independently identified eligible studies, extracted the data, and evaluated the quality of the literature. Pooled risk ratios (RRs) or weighted mean differences with 95% confidence intervals were calculated using either fixed or random effects models. Descriptive analysis was used when data could not be pooled.

**Results:**

A total of six studies were included in the review. For the revision percentage and complications, there were no significant differences between 2G PFA and TKA (RR = 2.29, 95% CI 0.69–7.58, P = 0.17; RR = 0.56, 95% CI 0.23–1.40, P = 0.22, respectively). Second, the results demonstrated that the differences in the Oxford Knee Score (OKS) and the University of California, Los Angeles (UCLA) activity score between 2G PFA and TKA were not significant (WMD −4.68, 95% CI −16.32 to 6.97, p = 0.43; WMD 0.16, 95% CI −1.21 to 1.53, P = 0.82). The Knee Injury and Osteoarthritis Outcome Score (KOOS), the American Knee Society Score (AKSS), and the Western Ontario and McMaster Universities Osteoarthritis Index (WOMAC) were presented in a narrative form due to methodological heterogeneity.

**Conclusion:**

For isolated PFOA, 2G PFA demonstrated similar results to TKA with respect to the percentages of revisions, complications, and PROMs.

## Introduction

Knee osteoarthritis (KOA) is a common type of degenerative disease in middle-aged and elderly individuals worldwide [1]. The knee consists of two articulations, the tibiofemoral (TF) and patellofemoral (PF) joints, in which osteoarthritis (OA) can occur in isolation or in combination [2]. To date, most related research has focused on the TF joint, in which total knee arthroplasty (TKA) has been proven to be a reliable treatment. However, the prevalence of isolated patellofemoral osteoarthritis (PFOA) has been estimated to be as high as 21%, and the percentage of patients with obvious clinical symptoms is up to 8% [3, 4]. The optimal surgical option for severely isolated PFOA remains controversial. Compared with TKA, patellofemoral arthroplasty (PFA) preserves the structural integrity of the tibiofemoral joint by sparing the condylar surfaces, menisci, and cruciate ligaments and is purported to permit more natural knee movement and proprioception [5]. Therefore, it was once considered an alternative to TKA in patients with isolated PFOA. Despite the theoretical advantages, TKA is still used for isolated PFOA, as PFA has been questioned due to its high percentages of failure and inconsistent results [6].

In the past few decades, 2G PFA, which has a more accurate anatomic design that attempts to better mimic patellofemoral joint function, has been introduced [7]. Studies [5, 8] have proven sustained improvements in functional outcomes and longer survivorship with new implants using this technique. Moreover, a few studies have been designed to directly compare 2G PFA with TKA. In addition to revisions and complications, PROMs have also been used to evaluate the effect of PFA in these studies [9, 10]. Therefore, the aim of this study was to compare the percentages of revisions, the complications, and the PROMs following the use of 2G PFA and TKA in treating isolated PFOA based on comparative studies.

## Methods

### Search strategy

We performed the current systematic review in accordance with the standards of the Preferred Reporting Items for Systematic Reviews and Meta-Analyses (PRISMA) in reporting the findings of this review [11]. Two researchers independently searched MEDLINE, Embase, and Web of Science to identify studies that compared the outcomes of PFA and TKA in the treatment of isolated PFOA. The three electronic databases were searched on February 15, 2021, and no time limitation was applied. The following keywords or corresponding Medical Subject Headings (MeSH) were used: “(patellofemoral OR PFA) AND (total knee OR TKA) AND (arthroplasty OR replacement) AND patellofemoral osteoarthritis AND (outcome OR revision OR complication OR patient-reported outcome measures).” The references of related studies (especially reviews and meta-analyses) on PFA versus TKA were also carefully screened to identify studies that were not captured in our initial database search. There were no language restrictions.

### Inclusion and exclusion criteria

The inclusion criteria are listed as follows: (a) randomized and nonrandomized trials, prospective and retrospective cohort studies, and case-control studies; (b) studies investigating the use of 2G PFA or TKA for isolated PFOA; (c) reports on results with revisions and complications; and (d) reports on PROMs with either the Oxford Knee Score (OKS), the Knee Injury and Osteoarthritis Outcome Scores (KOOS), the American Knee Society Score (AKSS), the Western Ontario and McMaster Universities Osteoarthritis Index (WOMAC), or the University of California, Los Angeles (UCLA) score.

The exclusion criteria were as follows: (a) duplicate articles; (b) case reports, reviews, meta-analysis, and cadaver experiment studies; (c) studies whose full text was not available; (d) studies with data that could not be extracted; and (e) reports that were not relevant to this study.

### Quality assessment

The quality of the studies was assessed by two reviewers independently using the Cochrane Collaboration Risk of Bias Assessment Tool [12] for assessing randomized controlled trials (RCTs) and the GRADE (grading of recommendations, assessment, development and evaluation working group) tool [13] for nonrandomized studies. The quality items assessed were random sequence generation, allocation concealment, blinding of participants, personnel and outcome assessors, completeness of outcome data, selective reporting, and other sources of bias. Each item was assessed as adequate (i.e., low risk of bias), inadequate (high risk of bias), or unclear (uncertain risk of bias). The methodological quality of the selected studies was classified by the overall summary assessment of all key items.

### Data extraction

Two researchers independently reviewed and extracted data from the included studies. The following data were extracted from each study: first author’s name, date of publication, sample size, study design, intervention and prosthesis type, follow-up time, outcomes (patient-reported outcome measures, revisions, and other complications), and knees available at the final follow-up. An experienced third researcher helped resolve any divisions between the two authors.

### Statistical analyses

Assessment of heterogeneity between the included studies was conducted to evaluate the feasibility of the meta-analysis. If the data could not be pooled due to clinical or methodological heterogeneity, the outcomes are presented as the mean with standard deviation along with p-value in a narrative manner. RevMan 5.3 software provided by the Cochrane Collaboration Network was used to conduct the statistical analysis, and the heterogeneity between the studies was calculated by the Q-test and I^2^ test. ① If p > 0.1 or I^2^ ≤ 50%, we considered there to be no obvious heterogeneity between the included studies and used a fixed effects model to pool the data. ② If p < 0.1 or I^2^ > 50%, we considered there to be heterogeneity among the results, and the random effects model was used to combine the data and analyze the sources of the heterogeneity. For continuous variables, we use the mean difference (MD) with 95% confidence interval (95% CI); for categorical variables, we use the risk ratios (RR) with 95% confidence interval (95% CI). All p-values were two-sided. A p-value < 0 .05 was considered statistically significant.

## Results

### Description of studies

**Fig. 1:**
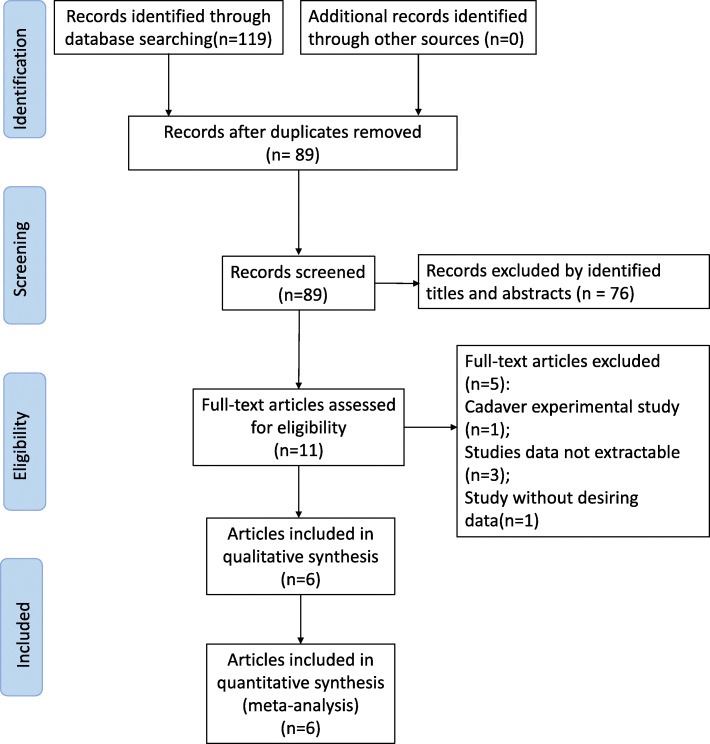
Flowchart of the literature search in the meta-analysis

A total of 119 studies were identified in the literature search, 30 of which were excluded as duplicates. After primarily reading the article titles and abstracts, 78 articles were excluded for not meeting the inclusion criteria, and 5 studies were excluded according to the exclusion criteria (one cadaver-based study, 3 studies without full text available, and one study without desired data). Therefore, six studies, including 2 RCTs [14, 15], 2 retrospective cohort studies [16, 17], and 2 retrospective case-matched studies [18, 19], were included for systematic review and meta-analysis. The 2018 study by Perrone et al. has three study arms: one group received PFA, one group received TKA with patelloplasty, and a third group served as a control group undergoing TKA with patella resurfacing. We excluded the TKA with patelloplasty arm of the study. A total of 16 patients were excluded due to this process. We were then left with a total of 397 patients, 201 of whom underwent PFA and 196 who underwent TKA as controls. Our literature search process is shown in Fig. 1, and the characteristics of the included studies are presented in Table 1.

**Table 1:** Characteristics of included studies

Study	Sample size	Study design	Intervention	Control	Outcomes assessed	Outcomes measure	Length of follow-up	Knees available at final follow-up
Joseph et al. [14]	64 (32/32)	RCT	Avon (Stryker)/FPV (Wright Medical)/Zimmer (Zimmer Biomet) for PFA	NexGen (Zimmer Biomet)/Vanguard (Zimmer Biomet)/Triathlon (Stryker)/Medial Pivot (MicroPort orthopedics) for TKA	WOMAC, OKS, AKSS, UCLA, complications	WOMAC, OKS, AKSS, UCLA, complications	60 months	43 (20 TKA; 23 PFA)
Odgaard et al. [15]	100 (50/50)	RCT	Avon (Stryker) for PFA	PFC (DePuy) for TKA	OKS, KOOS, SF-36, complications	OKS, KOOS, SF-36, complications	2 years	93 (47 TKA; 46 PFA)
Dahm et al. [16]	45 (23/22)	Retrospective cohort study	Avon (Stryker) for PFA	Zimmer (Warsaw)/SIGMA (DePuy) for TKA	AKSS, UCLA, KSS, satisfaction, complications	AKSS, UCLA, KSS, satisfaction, complications	29 months for PFA, 27 months for TKA	45 (23 PFA; 22 TKA)
Clement et al. [19]	108 (54/54)	A retrospective case-matched cohort	Avon (Stryker) for PFA	Triathlon (Stryker) for TKA	OKS, SF-12, satisfaction, complications	OKS, SF-12, satisfaction, complications	9.2 years	87 (41 PFA; 46 TKA)
Perrone et al. [17]	34 (19/15)	Retrospective cohort study	PFJ (Zimmer) for PFA	The NexGen LPS (Zimmer Biomet) for TKA	OKS, KOOS, Kujala Score, complications	OKS, KOOS, Kujala Score, complications	More than 3 years	34 (19 PFA; 15 TKA)
Kamikovski et al. [18]	46 (23/23)	A retrospective case-matched cohort	Avon (Stryker)/PFJ(Zimmer) for PFA	TKA	KOOS, WOMAC, UCLA	KOOS, WOMAC, UCLA	5.2 years for PFA; 5.4 years for TKA	46 (23 PFA; 23 TKA)

### Risk of bias in the included studies

**Fig. 2:**
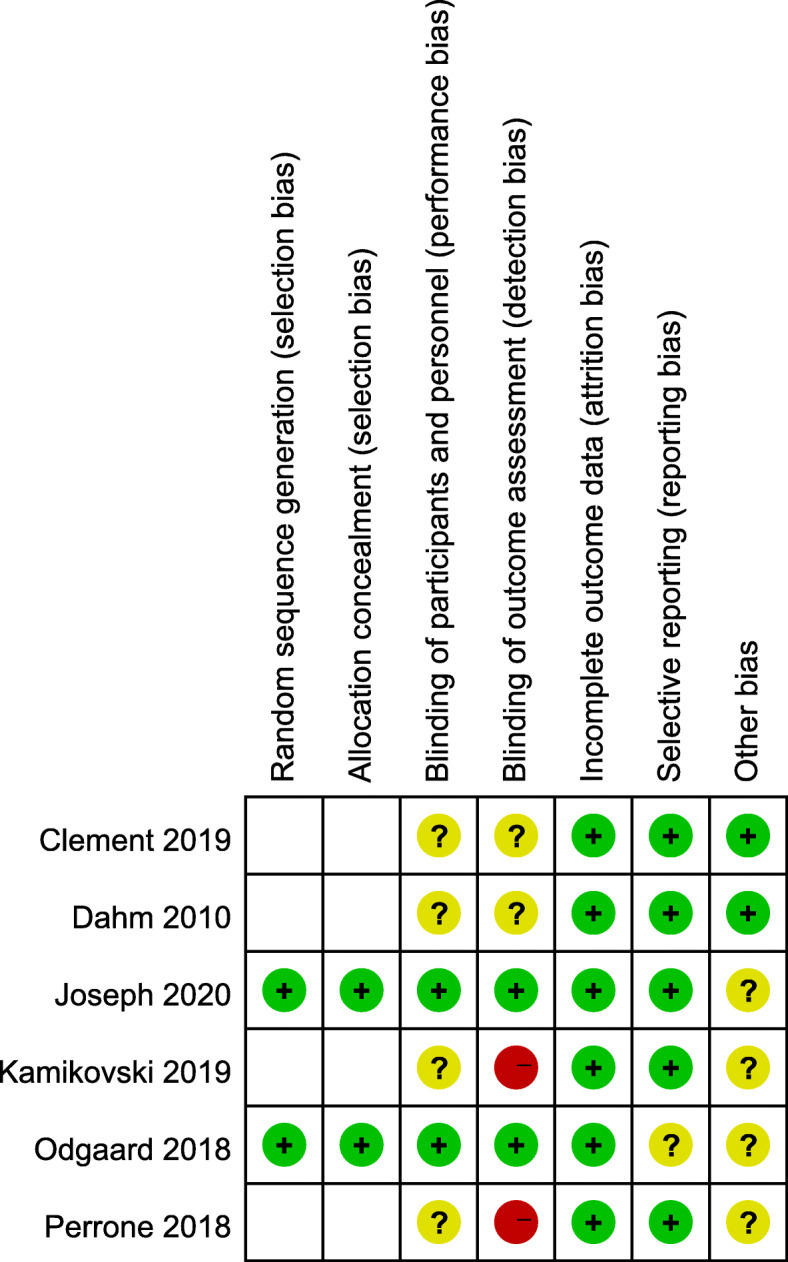
Risk of bias for included studies

Overall, the methodological quality of the included trials was not high and varied substantially. Only two studies were graded as high quality for having no more than two items with an uncertain risk of bias, two studies were graded as unclear quality, and two studies were graded as low quality for having one key item with a high risk of bias (Fig. 2).

### Outcome analysis

#### The Oxford Knee Score (OKS)

**Fig. 3:**
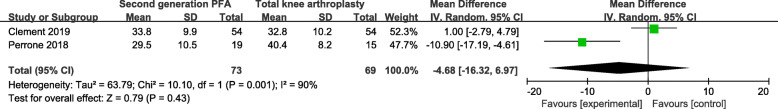
Forest plot on the assessment of OKS

Four of the included studies [14, 15, 17, 19] evaluated patients with OKS. Two studies [17, 19] with 142 participants reported the total OKS score (0–48), where a higher score indicated better function. In contrast, Joseph et al. [14] defined a lower OKS score as better function and reported no statistically significant difference between the PFA and TKA groups at 24 and 60 months postoperatively (15.4 ± 9.2 vs 13.7 ± 8.33, P = 0.392; 12.1 ± 9.2 vs 13.5 ± 9.2, P = 0.596, respectively). Odgaard et al. [15] compared the differences in the area under the curve at the group level, which was calculated based on improvements in the postoperative OKS. No statistically significant difference between the PFA and TKA groups was found in their study at the 2-year follow-up (p = 0.282). Due to the above methodological heterogeneity, only two studies [17, 19] were used to perform the meta-analysis. The pooled data demonstrated no significant differences regarding the OKS between the PFA and TKA groups at the last follow-up (WMD −4.68, 95% CI −16.32 to 6.97, p = 0.43). The forest diagram is shown in Fig. 3.

#### The University of California, Los Angeles (UCLA) activity score

**Fig. 4:**
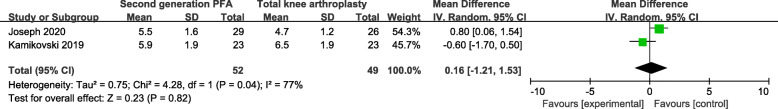
Forest plot on the assessment of UCLA activity

Three studies [14, 16, 18] used the UCLA activity score to evaluate the outcomes. Dahm et al. [16] found a higher mean postoperative UCLA activity score in the PFA group than in the TKA group (6.6, range 5 to 9, vs 4.2, range 3 to 6, P < .0001). The pooled data of the other two studies [14, 18] revealed no significant difference regarding the UCLA activity scores between the two groups (WMD 0.16, 95% CI −1.21 to 1.53, P = 0.82); the forest diagram is shown in Fig. 4. Moreover, Joseph et al. [14] also reported the UCLA Walking and Function scores, but neither of these was found to be different between the PFA and TKA groups (8.6 ± 2.3 versus 8.0 ± 2.1, p = 0.425; 8.0 ± 2.4 versus 6.9 ± 3.0, p = 0.425, respectively).

#### Western Ontario and McMaster Universities Osteoarthritis Index (WOMAC)

Two of the included studies [14, 18] used the WOMAC to evaluate the patients postoperatively. Joseph et al. [14] reported that a lower total WOMAC indicated better function, and their results indicated that there were no statistically significant differences between the PFA and TKA groups at 3, 6, and 12 months postoperatively (P > 0.05). However, Kamikovski et al. [18] reported the total WOMAC using the opposite definition. They reported that TKA performed better than PFA at 1 year in terms of the total mean WOMAC (63.5 ± 28.0 vs 83.0 ± 13.9, p = 0.035), but the groups did not have significantly different scores at 2 years (77.1 ± 18.7 vs 85.2 ± 15.5, p = 0.157).

#### Knee Injury and Osteoarthritis Outcome Scores (KOOS)

Three studies [15, 17, 18] reported KOOS-related results. Perrone et al. [17] used the Knee Injury and Osteoarthritis Outcome Score—Physical Function (KOOS-PS), which is derived from the original and extended form of the KOOS. The total values of the KOOS-PS ranged from 0 (absence of problems) to 28 (patients report severe problems). Finally, no significant difference regarding the KOOS-PS was found between the PFA and TKA groups (15.3 ± 7.1 vs 12.9 ± 5.1, p = 0.509) after a mean time of 32.7 months postoperatively. Odgaard et al. [15] compared the improvement between the baseline and the follow-up of the two groups. The improvement in every subscale of the KOOS (symptoms, pain, function of daily living, sports and recreation, and quality of life) was calculated. The results showed that PFA was better than TKA regarding symptoms and sports and recreation (P < 0.05) at the 1-year follow-up, but except for symptoms, none of the subscales showed statistically significantly different scores after 2 years (P > 0.05). Kamikovski et al. [18] reported different results: TKA performed better than PFA after 1 year for the KOOS subscales pain, activities of daily living, and sports and recreation (P < 0.05), but the groups did not have significantly different scores after 2 years (P > 0.05).

#### American Knee Society Score (AKSS)

Two of the studies [14, 16] reported the AKSS knee score and AKSS function score. Because of the clinical and methodological heterogeneity, we performed a narrative analysis instead of a meta-syntheses. Joseph et al. [14] showed that neither of the scores differed between the PFA and TKA groups at 12 months postoperatively (76.3 ± 15.8 versus 77.4 ± 18.8, P = 0.847 for AKSS knee score; 77.3 ± 17.9 vs 73.9 ± 19.7, P = 0.532 for AKSS function score). Dahm et al. [16] used the mean and range instead of the standard difference to report the outcomes, in which the mean postoperative AKSSs were 89 (range 69 to 100) and 90 (range 47 to 100) for the PFA and TKA groups, respectively (P = 0.85), and the mean postoperative AKSS function scores were 84 (range 51 to 100) in the PFA group and 73 (range 59 to 94) in the TKA group (P = 0.05).

#### Revisions

**Fig. 5:**
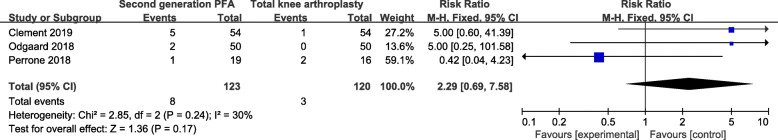
Forest plot on the assessment of revision

Three studies [15, 17, 19] reported the number of revisions, noting a total of 8 in the PFA group and 3 in the TKA group during the follow-up period. However, our meta-analysis demonstrated that there was no significant difference between the two groups (RR = 2.29, 95% CI 0.69–7.58, P = 0.17). The forest diagram of revisions is shown in Fig. 5.

#### Complications

**Fig. 6:**
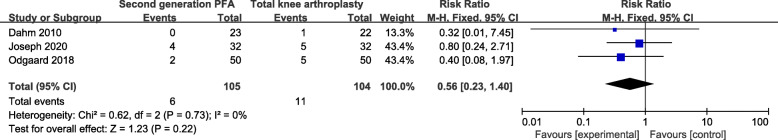
Forest plot on the assessment of complications

Three studies [14–16] analyzed operation-related complications. Joseph et al. [14] reported four superficial infections in the PFA group and five in the TKA group. Dahm et al. [16] found no significant complications in the PFA group, but noted that one case of deep vein thrombosis occurred in the TKA group. Odgaard et al. [15] indicated that two patients who underwent PFA and four patients who underwent a combined five TKA procedures experienced patellar instability. The pooled data demonstrated that there was no significant difference between the two groups regarding operation-related complications (RR = 0.56, 95% CI 0.23–1.40, P = 0.22). The forest diagram of revisions is shown in Fig. 6.

## Discussion

Recently, the use of PFA to treat isolated PFOA has aroused interest again given the improvements in the newer designs [20]. However, there is a dearth of multicenter RCTs directly comparing 2G PFA with TKA for isolated PFOA. As a result, we decided to perform this systematic review and meta-analysis based on RCTs and comparative studies. The significant findings were that both procedures are comparable regarding the revision percentages, complications, and short-term PROMs.

In terms of revision percentages, our findings indicated no significant differences between the two operative options, which is different from previous opinions [21, 22]. In the past few decades, many surgeons have been reluctant to use PFA due to its high failure and revision percentages. However, the most common causes of revision for these patellofemoral implants reported in past studies were progression of tibiofemoral OA and prosthetic problems, which accounted for more than 50% of the revision after 15 years of follow-up [23–25]. Some authors further reported that tibiofemoral OA progression is more frequent in obese patients and when the indication is primary PFOA than in those affected by trochlear dysplasia [26, 27]. Therefore, we believe that given the improvements in the new prostheses, the incidence of revisions in 2G PFA may, in part, be due to challenges related to patient selection. Our results are in accordance with other studies. Cartier et al. [28] reported longer-term results with the Richards prosthesis (used in 2G PFA) and demonstrated that most patients continued to demonstrate good function after a decade. A meta-analysis of 28 studies by Dy et al. [29], comparing complications of PFA and TKA for isolated PFOA, found an 8-fold higher likelihood of revision for all PFAs relative to TKA. However, when only 2G PFA was compared, no significant differences in reoperations, revisions, or complications were found. Moreover, several authors confirmed that PFA could potentially delay TKA by 10 to 15 years in up to 80% of patients, and revision to TKA can be performed without difficulty [25, 30–32]. Hence, we believe that 2G PFA is an acceptable option for isolated PFOA as long as patients are selected appropriately.

Regarding PROMs, we compared most associated parameters, including the OKS, AKSS, WOMAC, and UCLA score. The recognition of incongruity between patient-based and surgeon-based evaluations after surgical treatment has led to an increasing utilization of PROMs as an outcome evaluation method, and they are now recommended as an important outcome measure and used extensively when evaluating patients undergoing joint replacement surgery. Given the lack of uniformity in the included articles, we only undertook a meta-analysis of the OKS and UCLA score. First, for the OKS, Bunyoz et al. [33] reported that a weighted mean OKS of 36.7 was found in the 2G PFA group in a systematic review, which was categorized as a good outcome (>34) and is comparable with scores found in other studies investigating the outcome of unicompartmental knee arthroplasty. Although all the mean OKS scores of 2G PFA in our studies were lower than those in the Bunyoz et al. study, our meta-analysis also demonstrated a good outcome in which PFA was comparable to TKA.

The UCLA scale is a simple scale ranging from 1 to 10, with level 1 defined as “no physical activity, dependent on others” and level 10 defined as “regular participation in impact sports” [34]. Recent attention has focused on patient activity levels after arthroplasty. The pooled data showed no significant difference between the two groups (P = 0.82), which indicated no difference in the activity level following the two procedures. Chang et al. [35] found that worse postoperative UCLA activity scores were associated with no regular sports activity after TKA. They further noted that the reasons these activities were impaired were not limited to the replaced knees alone. Symptoms related to the spine or the nonreplaced knee accounted for 25% and 8%, respectively. Their findings can be partially explained by the fact that the process of osteoarthritis can involve multiple joints [36]. However, patients undergoing PFA should be younger than those receiving TKA; therefore, most patients with isolated PFOA are seldom affected by osteoarthritis of other joints in the short term. Therefore, PFA is expected to achieve a better activity level than TKA.

In addition, we performed a narrative synthesis of the AKSS, KOOS, and WOMAC due to the presence of clinical, methodological, and statistical heterogeneity. The outcomes after PFA were either similar or better than those after TKA at the short-term follow-up. Bunyoz et al. [33] published a systematic review with the primary aim of comparing the outcomes of second-generation PFA and TKA by the assessment of PROMs. Their results showed that the weighted mean AKSS knee score was over 80 in both the 2G PGA and TKA groups without a significant difference. In a prospective study by Cotic et al. [37], 29 patients were treated with PFA, and the 2-year follow-up results indicated significant improvements (more than 20, p < 0.05) in the WOMACs when compared with the baseline values. These results showed that PFA is noninferior to TKA in the short term. However, it is difficult to draw a conclusion regarding which procedure is better due to the uniformity and the lack of a meta-analysis.

Previous studies have found that early complications such as persistent anterior knee pain, patellar catching or snapping, intraoperative fracture, and extensor mechanism failure are more frequent in PFA than in TKA and are mostly related to malpositioning. Other specific complications, including lateral swelling, patellar maltracking or instability, peripatellar pain, and lateral catching, have also been mentioned. However, the results of this study show that 2G PFA does not increase the percentage of complications, and the above complications were less common in our included articles, which may be attributed to the improvements in the new implants. The newly designed implants have evolved substantially, with the ability to externally rotate and translate the position of the femoral trochlea component, thus reducing the loads on the lateral facet. There is a broad and relatively unconstrained trochlea in the appropriate position, allowing the patella button to be captured and stabilized within the groove as flexion occurs [5]. Therefore, we assume 2G PFA to be an effective procedure with fewer complications and more rapid recovery after surgery.

We acknowledge this study still has some limitations that need to be further explored. First, the heterogeneity among the included studies makes it impossible to conduct a meta-analysis of every parameter; therefore, inaccuracy is introduced in the reporting of data as a narrative synthesis. Second, the low level of evidence in the studies introduces a considerable risk of selection bias in the studies, which poses a threat to the validity of our results. Third, most of the studies included focus on short-term follow-up outcomes, preventing long-term efficacy comparisons. Fourth, patellar resurfacing is usually performed during TKA, but it is not mentioned in detail in all of the included studies, which might cause a possible bias.

## Conclusion

For isolated patellofemoral osteoarthritis, compared with TKA, 2G PFA demonstrated similar results with respect to revision percentages, complications, and PROMs. We believe that with appropriate patient selection, PFA is a comparable option for the treatment of isolated PFOA in the short term. Further research is warranted to evaluate the long-term results of second-generation PFA.

## Data Availability

The datasets used and/or analyzed during the present study are available from the corresponding author on reasonable request.
